# Myocardial extracellular volume measurement using cardiac computed tomography

**DOI:** 10.1007/s10554-024-03226-4

**Published:** 2024-10-14

**Authors:** Rahul G. Muthalaly, Timothy Abrahams, Andrew Lin, Kush Patel, Sean Tan, Damini Dey, Donghee Han, Balaji K. Tamarappoo, Stephen J. Nicholls, Nitesh Nerlekar

**Affiliations:** 1 Victorian Heart Institute, Monash University, 631 Blackburn Road, Clayton, VIC 3168 Australia; 2 Victorian Heart Hospital, Monash Health, Clayton, VIC Australia; 3 St. Bartholomew’s Heart Centre, London, UK; 4 Department of Biomedical Sciences, Biomedical Imaging Research Institute, Cedars-Sinai Medical Centre, Los Angeles, CA USA; 5 Cardiovascular Institute, Indiana University School of Medicine, Indianapolis, IN USA; 6 Baker Heart and Diabetes Institute, Commercial Road, Melbourne, 3004 Australia

**Keywords:** Fibrosis, Myocardium, Multidetector Computed Tomography, Cardiomyopathies

## Abstract

Myocardial fibrosis is a common endpoint of many cardiac diseases and increasingly recognized as a predictor of heart failure, arrhythmia, and death. Recent studies have utilised cardiac computed tomography (CT) scans with delayed phase imaging to quantify diffuse fibrosis of the myocardium. CT extracellular volume (CT-ECV) measurement correlates well with CMR and histological myocardial fibrosis. Furthermore, CT-ECV predicts outcomes such as death, heart failure and arrhythmia in various disease states. This review summarizes the rationale and methodology behind CT-ECV measurement and provides a detailed summary of the current clinical evidence for the use of CT-ECV.

## Graphical abstract

**Figure:**
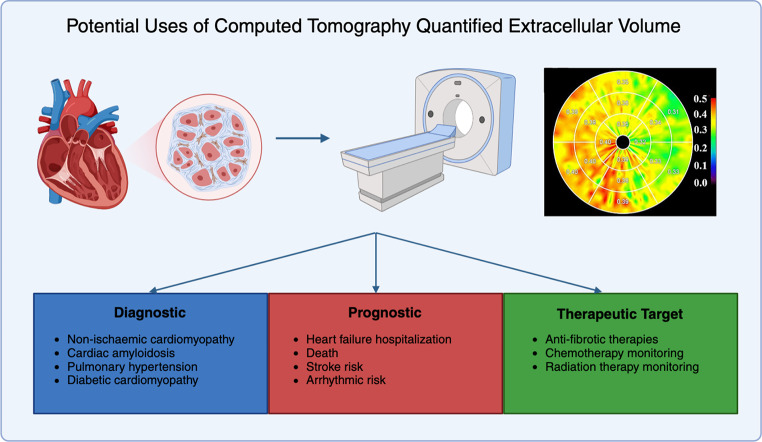
Cardiac Computed Tomography Extracellular Volume Assessment (CT-ECV) offers rapid and accurate quantification of myocardial fibrosis. This modality requires additional delayed imaging which allows for assessment of iodine concentrations in the myocardium. This concentration correlates to the degree of myocardial fibrosis. CT-ECV can be used to identify myocardial disease and prognosticate for future risk of cardiovascular outcomes

## Background and principles of myocardial fibrosis imaging

**Fig. 1:**
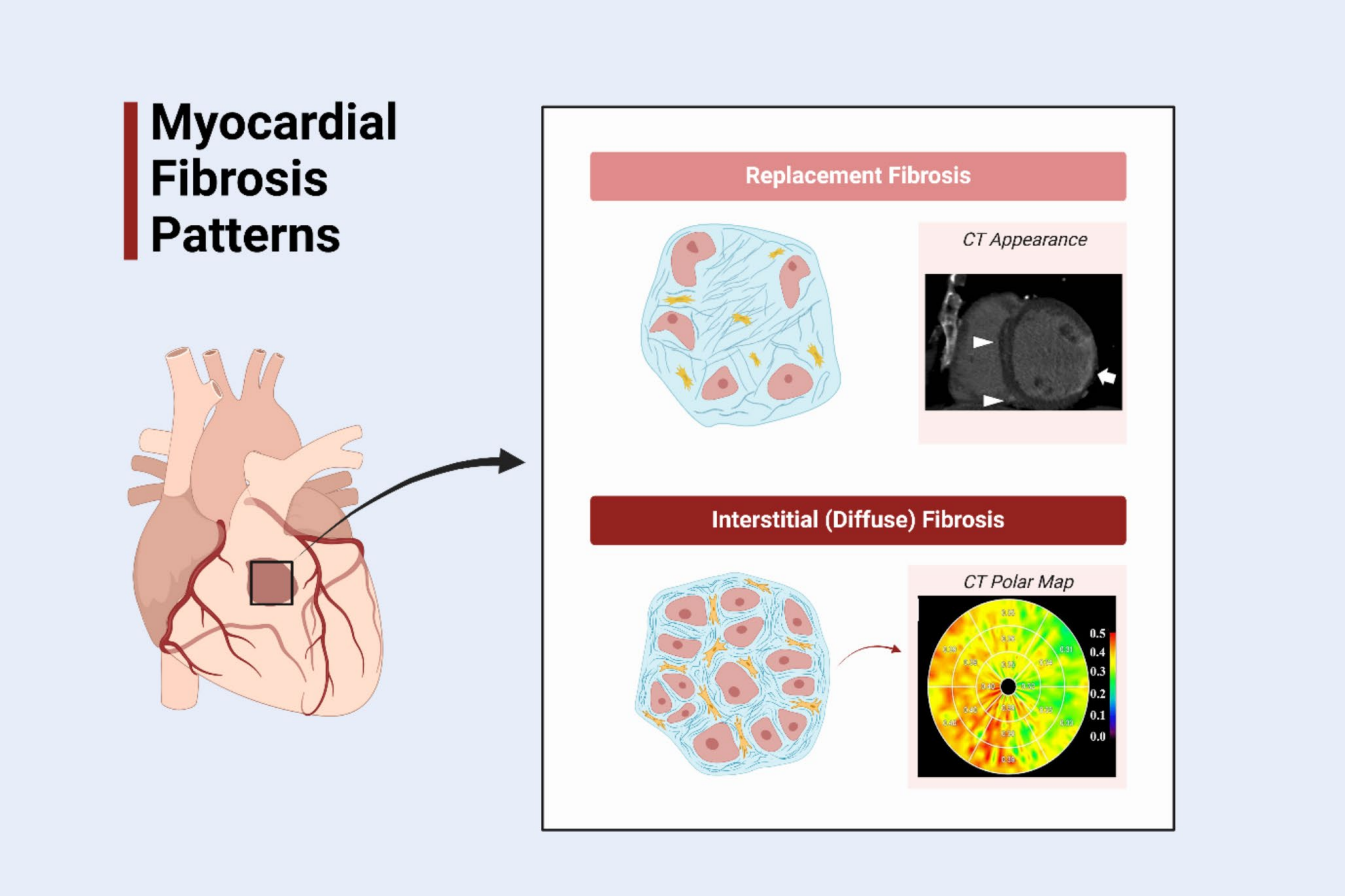
Different Patterns of Myocardial Fibrosis. Illustration demonstrating different types of myocardial fibrosis. Replacement fibrosis occurs after myocyte necrosis due to an insult (usually myocardial infarction). This results in replacement of those cells with a network of collagens and other extracellular proteins. Replacement fibrosis can be detected on delayed phase CT as shown. In interstitial (diffuse) fibrosis extracellular proteins are deposited without the loss of myocytes leading to myocardial stiffness. This can be quantified using cardiac computed tomography as shown in the polar map

Fibrosis involves the deposition of extracellular matrix (ECM) proteins in tissue, usually due to activated repair mechanisms in response to chronic injury. Myocardial fibrosis is a common pathologic endpoint for several cardiac diseases and can occur with ageing [1]. Diffuse myocardial fibrosis arises due to expansion of collagen in the interstitial space surrounding myocytes as shown in Fig. 1 [2]. Expansion of the interstitium can also occur with deposition of other molecules, such as amyloid (in amyloidosis) and sphingolipids (in Fabry disease) [3]. As interstitial fibrosis accumulates, progressive myocardial dysfunction occurs with a pattern that typically affects diastole followed by systole [4].

CMR offers accurate and non-invasive assessment of myocardial fibrosis using gadolinium, a heavy metal extracellular contrast agent, which increases signal intensity on T1-weighted images. Gadolinium remains in the extracellular space for up to 15 min after bolus dosing. Post-contrast T1-maps can be created using CMR allowing derivation of the extracellular volume fraction (ECV) of the myocardium after comparison to the blood pool (see formula 1). ECV (composed of the vascular and interstitial spaces) in a healthy heart ranges between 23 and 28% of the myocardial volume [5]. CMR T1-mapping is accurate when compared to histology and can identify disease processes and prognosis in non-ischaemic cardiomyopathies such as cardiac amyloidosis or Fabry’s disease [6].

1 $$\:{ECV}_{CMR}=\:\frac{{\varDelta\:R}_{1T}}{{\varDelta\:R}_{1B}}\times\:(1-Haematocrit)$$

**Fig. 2:**
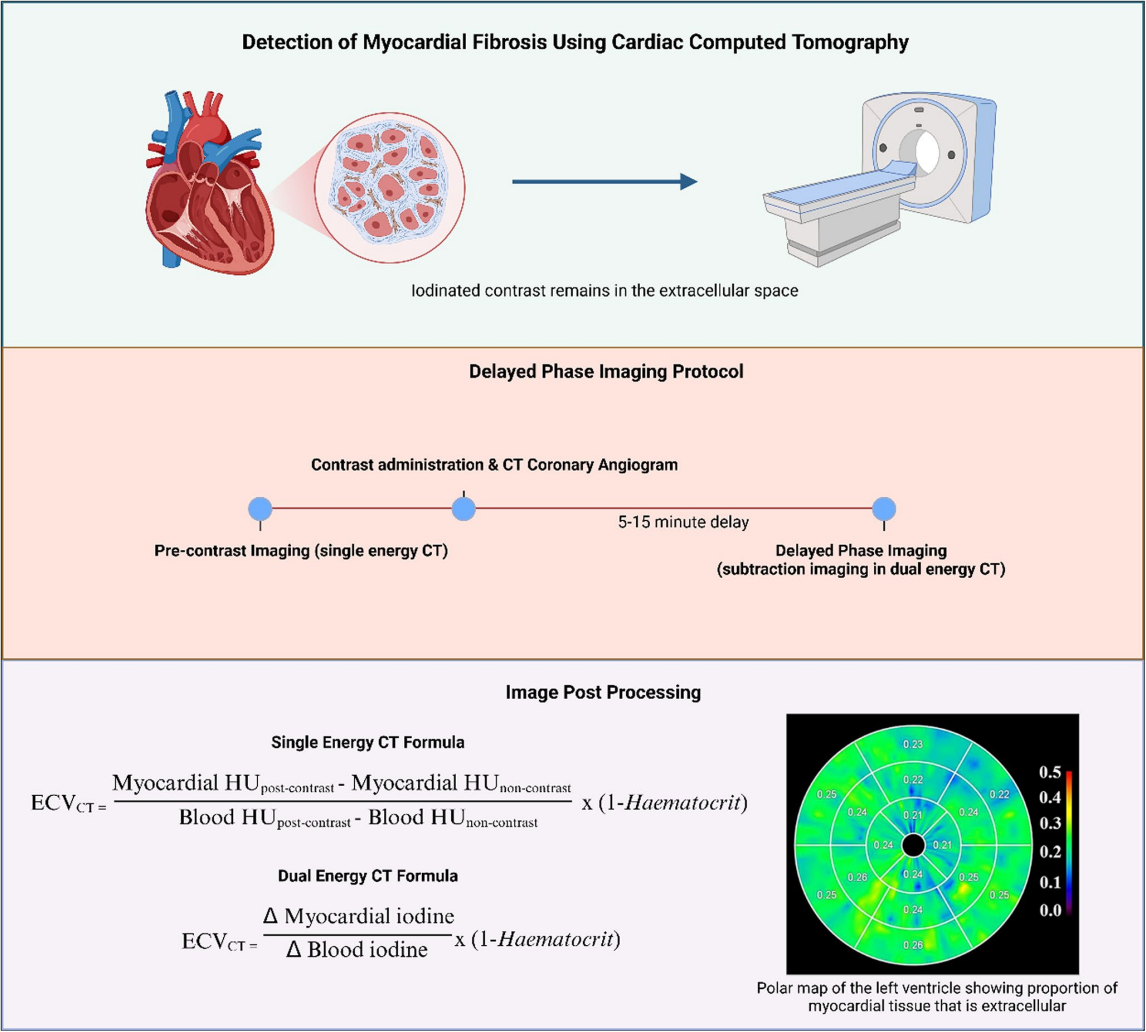
Methodology for Determining Extracellular Volume using Cardiac CT. Illustration demonstrating the methodology of determining myocardial extracellular volume fraction (ECV) from computed tomographic imaging (CT). With single energy CT, iodine contrast is administered followed by a delay of between 5 to 15 min to allow equilibrium between the blood and interstitial space. With dual energy CT, an iodine map is made using a single delayed post-contrast scan. Repeat CT image acquisition is performed with subsequent postprocessing according to the formula to determine the fraction of extracellular volume in the myocardial tissue

Recent work has demonstrated that ECV measurement using cardiac CT is accurate and reliable when compared to CMR and histology [7, 8]. CT-ECV is similar in principle to CMR: an extracellular contrast agent (iodine) is administered and diffuses from the vascular space into the interstitium. During the delayed phase, iodine remains in fibrotic tissue due to a low capillary density and dense ECM resulting in a higher density in Hounsfield units (HU). By comparing pre- and post-contrast images of the myocardium and blood pool after adjusting for the haematocrit (formula 2), one can establish the tissue concentration of iodine which correlates to the ECV fraction as shown in Fig. 2.

2 $$\begin{array}{l}\:EC{V_{CT}} = \:\frac{{Myocardial\:H{U_{post - contrast}} - \:Myocardial\:H{U_{non - contrast}}}}{{Blood\:H{U_{post - contrast}} - Blood\:H{U_{non - contrast}}\:}}\\\times \:(1 - Haematocrit)\end{array}$$

Nacif et al. compared CT-ECV to CMR T1-mapping in 24 subjects (13 heart failure and 11 healthy) and found that CT exams took only 13 min when compared to 47 min in CMR. Furthermore, there was excellent correlation between CMR-ECV and CT-ECV with *r* = 0.82 and bias of 3.01% for CT-ECV compared to CMR-ECV [7]. Excellent agreement between CMR-ECV and CT-ECV has been repeatedly demonstrated since then [9].

## How to perform CT extracellular volume assessment

**Fig. 3:**
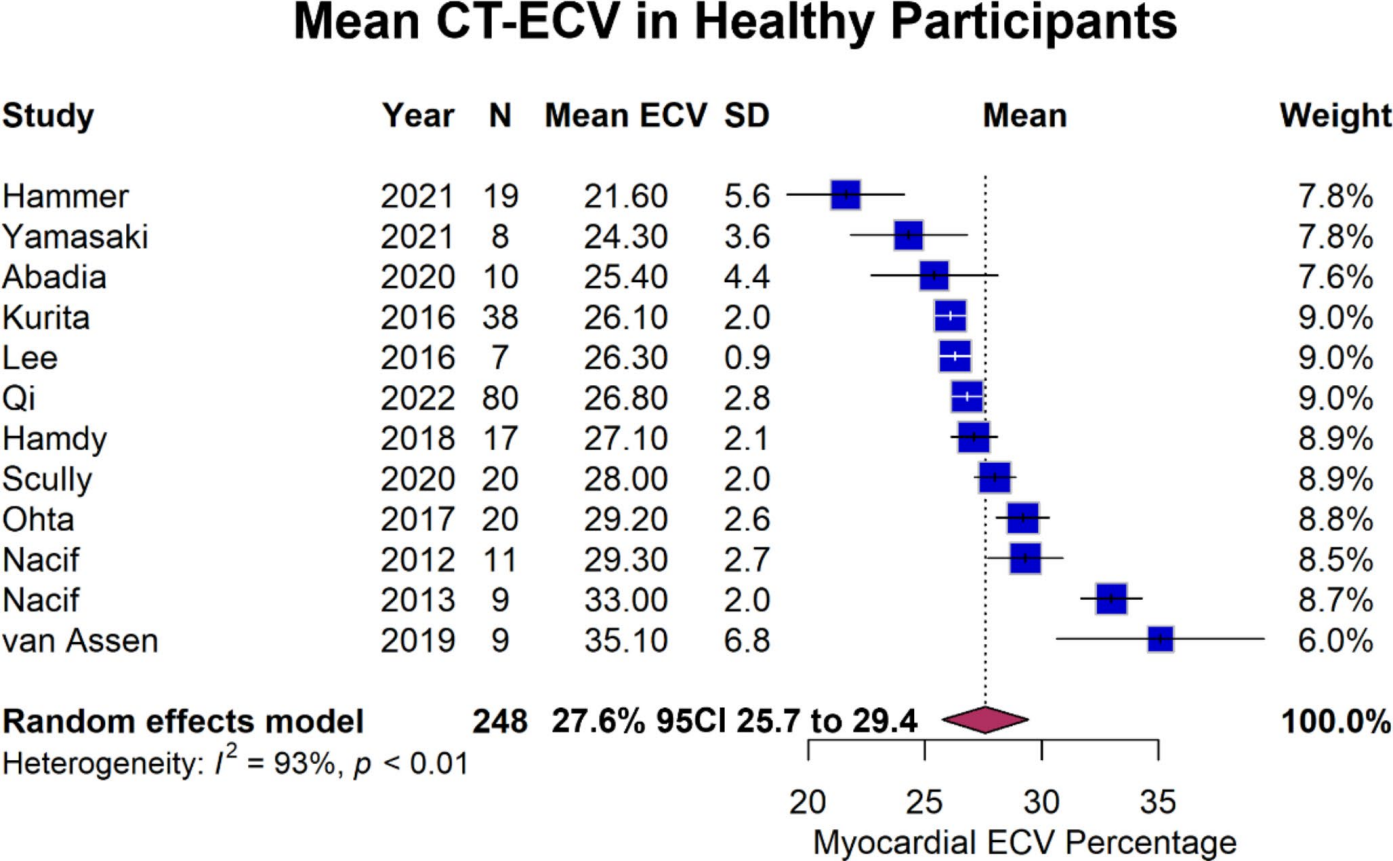
Forest Plot of Estimates of ECV Fraction in Healthy Individuals from a Range of Studies. Forest plot demonstrating CT determined extracellular volume fractions in healthy individuals across a range of studies. As can be seen, the I2 value is 93% suggesting high heterogeneity. Estimates in these studies span a large range from 21.6% to 35.1%. At least some of this difference is likely due to differences in CT-ECV protocols from factors such as delay time, post-processing methods, dual-energy versus single-energy CT and region of interest measured

There is no standardized protocol for CT-ECV quantification, making comparison difficult. A basic protocol consists of pre-contrast imaging followed by delayed phase imaging (3–7 min later) alongside haematocrit measurement **as shown in** Fig. 2. The use of dual energy CT scanners obviate the need for pre-contrast imaging as discussed below. There is significant variation across published protocols in the use of subtraction versus dual energy CT, timing of post-contrast scans, use of ‘synthetic’ or directly measured haematocrits, myocardial region of measurements and software. These differences may partially explain the variation in CT-ECV measurements in healthy controls in different studies which are shown in the highly heterogeneous forest plot in Fig. 3. We present a summary of available evidence around protocols and suggested parameters below.

### CT Acquisition parameters and contrast dose

CT acquisition parameters vary greatly in the CT-ECV literature, including slice thickness, reconstruction kernels and tube voltage. Most studies use an axial sequential mode with prospective ECG gating in keeping with usual cardiac CT protocols. Koike et al. examined the effect of high pitch helical scans when compared to axial sequential mode [10]. They found that the use of a high pitch mode was associated with an OR of 2.26 for sub-optimal image quality. We therefore suggest the use of axial sequential imaging.

Though reporting of kernels is poor across the literature, many studies use a smooth kernel to balance noise reduction while maintaining edge sharpness (for example, Siemens B20f) [11]. We believe that this is a reasonable choice, however, further data assessing the effect of kernels on reproducibility and agreement with CMR-ECV would be useful. Tube voltage and current also vary widely in the literature. One phantom study found higher variation in CT-ECV assessment at lower tube currents, particularly in the outer portion of images [12]. Further in vivo data is required examining the effect of tube voltage and current on CT-ECV quantification.

To date, most studies have reconstructed slice thickness at 3.0 to 8.0 mm to minimize noise while measuring the mean density in myocardial tissue. We support this approach while awaiting further data regarding the effect on CT-ECV accuracy [11, 13, 14].

There is a very wide variation in contrast dose protocols in CT-ECV studies to date, though all use low osmolality agents. Some studies use a fixed dose of contrast (for example 70mL or 100mL) while others use weight based regimens such 1mL per kg [15–17]. Arakawa et al. assessed the relationship between contrast dose and CT-ECV value in 95 patients [18]. They found that CT-ECV may be overestimated when a small dose of contrast is injected and that a dose of > 285 mg iodine/kg is needed to calculate CT-ECV. While this study was limited by the absence of a reference standard such as CMR or collagen volume fraction, the recommendation is reasonable to ensure accurate CT-ECV estimation. Unfortunately, contrast imaging in the setting of chronic kidney disease (such as that associated with amyloidosis where CT-ECV would be useful) remains challenging due to the risk of contrast-induced nephropathy. In this situation, pre-hydration with normal saline at 1mL/kg/hr for up to 6 h prior to contrast administration in addition to temporary cessation of exacerbating medications such as metformin and renin-angiotensin-aldosterone system blockers will minimize contrast induced nephropathy [19].

### Timing of Post-contrast Imaging

A delayed post-contrast acquisition is required to achieve contrast equilibrium between the plasma and interstitial spaces. The delay used in studies ranges from 3 to 15 min. Hamdy et al. assessed the optimal delay using signal-to-noise ratio and the consistency of ECV values over time. They found that a 5-minute delay achieved the highest signal-to-noise ratio, however, the CT-ECV values were consistent between 3, 5 and 7-minute delays in both normal myocardium and infarcted tissue [20]. Jablownoski et al. measured CT-ECV repeatedly in a swine model and found that equilibrium is reached 5 min after contrast administration and that ECV measurements remain consistent after that for up to 15 min [21]. Given these findings, we recommend a contrast bolus with a 5-minute delay to offer the optimal combination of signal-to-nose ratio and time efficiency.

### Dual energy versus single energy CT

Earlier studies relied on a subtraction method to define iodine concentration in the ECV of the myocardium. In this method, a pre-contrast gated scan is acquired to assess the baseline attenuation of the myocardium followed by a post-contrast scan which measures the attenuation in the same region of the myocardium. Dual energy scanners (DECT) utilise photons at two different energies to acquire two images with different properties. Iodine contrast attenuation varies greatly depending on photon energy, allowing for the creation of ‘iodine maps’ from a single acquisition. Iodine maps obviate the need for a pre-contrast scan which shortens the protocol and decreases the potential of misregistering the myocardium on the pre- and post-contrast scans. The subtractions and DECT methods produce similar results, with biases of much less than 1% [22]. If available, we recommend the use of a DECT protocol to shorten scan times and avoid misregistration.

## Region of myocardial measurement and analysis software

**Fig. 4:**
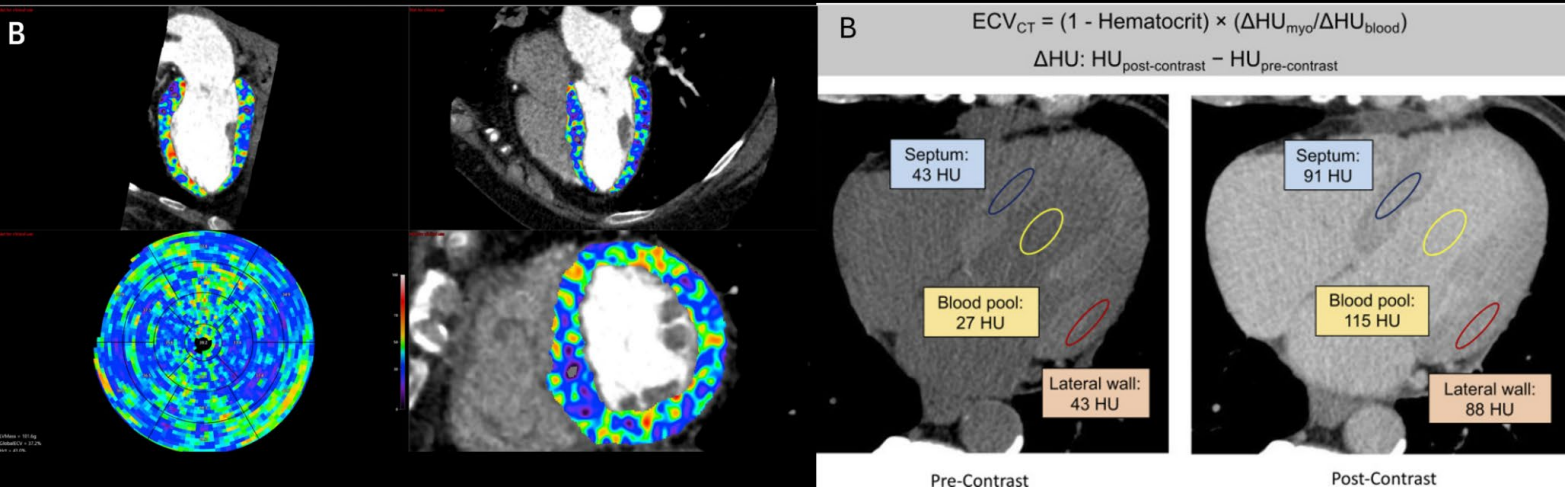
Global versus Local Measurement of Extracellular Volume using Computed Tomography. Images showing global versus local measurement of extracellular volume on CT. In (**A**) specialized software calculates CT-ECV values for each segment of the myocardium according to a 17-segment model which provides an understanding of regional variation in fibrosis. In (**B**) ROIs from the septum or lateral wall can be used to calculate local CT-ECV values. This does not require specialized software. The septum is often chosen to avoid beam hardening artifact from the spine. The formula used to calculate CT-ECV is demonstrated at the top of panel B

Studies investigating CT-ECV have used different of regions of interest (ROIs) to determine ECV fraction. Given that interstitial fibrosis is generally considered a diffuse process, many studies use a small ROI placed in the mid interventricular septum as a marker for the whole ventricle as shown in Fig. 4 [14]. The septum is chosen because of its distance from the vertebrae (which can produce beam hardening artifact) and the relative ease of identifying septal myocardium on non-contrast scans. Other studies have used a global measure with ROI placement in a 16- or 17-segment model [22] or in areas relevant to the disease process, such as the RV insertion point in pulmonary hypertension [23]. Clinically relevant differences in regional measurements have been recorded in studies [14]. Thus, we recommend using global measures where possible and reporting any segments with unusually high ECV. However, we recognize that segments may be affected by artifact due to factors such as implanted cardiac devices. In this situation, we suggest exclusion of those segments from the global ECV value. There may also be cases where segments are affected by clear late enhancement on CT, a feature which correlates highly replacement fibrosis identified on CMR [24]. Depending on the clinical circumstances, it may be appropriate to adopt a regional analysis approach and exclude the areas of late enhancement. For example, in a patient with known myocardial infarction but suspected concomitant amyloidosis, it would be appropriate to exclude the infarcted segments and calculated CT-ECV in the remainder of the myocardium. There is no head-to-head comparison of the prognostic value of CT delayed enhancement and CT-ECV.

There is a wide range of analysis software used in the literature. These include vendor specific software such as Syngo.via (Siemens) and Vitrea (Canon), independent software packages such as Ziosoft and custom designed 3D-ECV analysis software [14]. There has been no comparison of the reproducibility of these software packages to date.

### Synthetic versus direct haematocrit measurement

Traditionally, a recent haematocrit is required to calculate myocardial ECV fraction, so that a reference for iodine concentration in the blood pool after adjusting for blood cellularity can be established. There is reasonable variation in an individual’s haematocrit - reported as up to 12% over the course of 1–2 months [25]. This, in addition to the logistical barrier of blood drawing makes the idea of a ‘synthetic’ haematocrit derived from the imaging itself attractive. Treibel et al. validated a synthetic haematocrit using linear regression to estimate the haematocrit from the myocardial blood pool [26]. They demonstrated reasonable prediction and reliable CT-ECV estimation using this method, however, it has not been externally validated. We recommend institutions continue to use direct haematocrit measurements where possible until further synthetic haematocrit validation has been done.

## Clinical use cases of CT-ECV

CT-ECV has a rapidly growing body evidence supporting its utility in diagnosis and prognosis. With the widespread availability of cardiac CT, CT-ECV provides an opportunity to assess myocardial fibrosis in all patients undergoing coronary or structural heart evaluation for other reasons.

### Cardiac amyloidosis

**Fig. 5:**
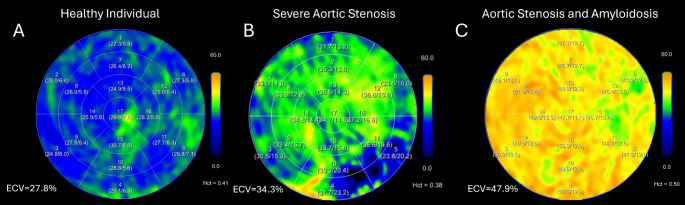
Bullseye Plots of Global CT-ECV Values in Different Cardiac Disease States. Bullseye plots of 17-segment models of left ventricles in (**A**) a normal individual with a mean CT-ECV of 27.8%, (**B**) an individual with severe aortic stenosis and a mean CT-ECV of 34.3% and (**C**) an individual with combined severe aortic stenosis and amyloidosis with a mean CT-ECV of 47

Cardiac amyloidosis develops due to deposition of amyloid protein in the extracellular space most commonly as a result of light chains from plasma cells (AL amyloidosis) or from misfolding of transthyretin protein (ATTR). Cardiac amyloidosis is more common than previously appreciated (15% of aortic stenosis 17% of HFpEF patients), with previous estimates limited by the poor availability of diagnostic testing [27]. The importance of identifying cardiac amyloidosis has become paramount with the availability of therapies that can attenuate the grave prognosis associated with the condition [28]. A hallmark of cardiac amyloidosis is extreme elevation of the ECV as shown in the bulls-eye plots in Fig. 5. Treibel et al. found a mean CT-ECV in cardiac amyloidosis is 54% when compared to 28% in patients with severe AS [11]. Subsequently, Kidoh et al. found that a CT-ECV cut off of 37% demonstrates 97% accuracy in diagnosing cardiac amyloidosis [29]. Thus, the routine implementation of CT-ECV as part of cardiac CT, for example in pre-TAVR work-up, may help to identify the 15% of patients with concomitant ATTR amyloidosis with only minimal impact on radiation and scan acquisition times [3].

### Valvulopathies

The feared endpoint of valvulopathies is deterioration of myocardial function due to structural changes, resulting from chronic adverse loading conditions. Severe AS results in increased afterload, which is initially compensated for by the development of left ventricular hypertrophy. CT-ECV can identify myocardial fibrosis which precedes the development of frank left ventricular dysfunction [30]. Cohort studies have verified the ability of CT-ECV to predict histological fibrosis in severe aortic stenosis patients as shown in Fig. 5 [8]. Cohort studies also demonstrates that high CT-ECV in severe AS patients undergoing valve replacement predict heart failure hospitalization, stroke and death [18]. Tamarappoo et al. found that 40% of severe AS patients with a CT-ECV of > 33% experienced death or heart failure when compared to just 9% of those below 33%. Early data also suggests that CT-ECV can predict myocardial recovery after transcatheter repair for mitral regurgitation [31]. Patients with a normal CT-ECV were more likely to recover their LV dimensions and systolic function whereas those with elevated CT-ECV failed to see improvement. Thus, we advocate for the routine use of CT-ECV in pre-procedural work up for valve replacement procedures.

### Non-ischaemic cardiomyopathy

Myocardial fibrosis is a common endpoint in non-ischaemic cardiomyopathies. It occurs as a result of myocardial injury due to the underlying pathological process, from inflammation to genetic anomalies. Abadia et al. demonstrated that CT-ECV is significantly higher in non-ischaemic cardiomyopathy when compared to healthy volunteers, with a cut-point of > 29.5% accurately predicting an underlying cardiomyopathy [16]. Further studies are needed to clarify the prognostic significance of an elevated ECV value, as well as exploring the diagnostic utility for differentiating between different cardiomyopathy types. However, we recommend CT-ECV as a useful add-on to initial coronary artery disease screening for newly identified cardiomyopathy to further understand myocardial health.

### Cardio-oncology

There is increasing recognition of cardio-toxicity form common cancer therapies including radiation, anthracyclines, and human epidermal growth factor receptor 2-directed therapies. These toxicities can have significant prognostic implications but current imaging markers to initiate cardioprotective therapies have been unhelpful. Cohort studies reveal that CT-ECV can identify early myocardial fibrotic changes in those undergoing anthracycline and radiation therapy [32, 33]. Some cancer surveillance imaging protocols may be able to integrate pre and post contrast cardiac imaging to allow for the CT-ECV calculation as part of routine follow-up. More work is needed to understand the clinical implications of CT-ECV changes during cancer therapy.

## Conclusions

CT-ECV represents an exciting new addition to the already high-value and rapidly expanding cardiac CT modality, extending its power beyond the coronaries. Early work has demonstrated utility in diagnosis and prognosis for cardiomyopathies. More research is needed regarding the optimal CT-ECV protocol and a study of its prognostic implications across the spectrum of cardiovascular disease. CT-ECV may also be an accessible and accurate end-point to assess the effectiveness of novel therapies targeting myocardial fibrosis.

## Data Availability

No datasets were generated or analysed during the current study.
